# Adaptive Memory: Independent Effects of Survival Processing and Reward Motivation on Memory

**DOI:** 10.3389/fnhum.2020.588100

**Published:** 2020-12-10

**Authors:** Glen Forester, Meike Kroneisen, Edgar Erdfelder, Siri-Maria Kamp

**Affiliations:** ^1^Department of Psychology, University of Trier, Trier, Germany; ^2^Department of Psychology, University of Koblenz-Landau, Landau, Germany; ^3^Department of Psychology, University of Mannheim, Mannheim, Germany

**Keywords:** survival processing, reward, event-related potentials, memory, motivation, encoding, P300, frontal slow wave

## Abstract

Humans preferentially remember information processed for their survival relevance, a memorial benefit known as the *survival processing effect*. Memory is also biased towards information associated with the prospect of reward. Given the adaptiveness of these effects, they may depend on similar mechanisms. We tested whether motivation drives both effects, with reward incentives that are known to boost extrinsic motivation and survival processing perhaps stimulating intrinsic motivation. Accordingly, we manipulated survival processing and reward incentive independently during an incidental-encoding task in which participants chose between pairs of words concerning their relevance for a scenario, and examined the effects on encoding event-related potentials (ERP) activity and later performance on a surprise recall test. We hypothesized that if survival processing fosters intrinsic motivation, it should reduce the beneficial effects of extrinsic motivation (reward incentive). In contrast to this prediction, we found that reward incentive and survival processing independently improved memory and that the P300, a measure of lower-level cognitive resource allocation, was increased by reward incentive independent of survival processing. Further, survival processing and reward incentive independently increased the frontal slow wave (FSW), a measure of higher-level elaboration. These findings suggest that while survival processing and reward incentive may both increase encoding elaboration, the memory-enhancing effect of survival processing does not depend on increased intrinsic motivation. Additionally, we replicated a recent finding whereby the survival processing effect generalizes to a choice-based encoding task and further showed that the beneficial effect of choice on memory likely does not interact with either survival processing or reward.

## Introduction

Memory has been described as “adaptive” from at least two perspectives. One perspective suggests that the memory system has evolved to preferentially encode and retrieve information potentially relevant to survival in ancestral conditions. Indeed, information encoded while imagining such conditions is better remembered, an advantage known as the *survival processing effect* (Nairne et al., 2007). The other perspective points out that our memory system is biased towards information associated with the anticipation or obtainment of reward (Shohamy and Adcock, 2010). Since such a bias was likely adaptive for our evolutionary ancestors, the two views on “adaptive memory” are complementary in principle, but the relationship between the memory-enhancing effects of survival processing and motivation for reward has not been systematically investigated so far. In the present study, we examined if survival processing and reward incentive improve memory through the same mechanisms by examining their separate and combined effects on memory recall and neural activity during memory encoding.

### A Role of Motivation in the Survival Processing Effect?

In the standard survival processing paradigm (Nairne et al., 2007), participants imagine that they are attempting to survive, alone and without provisions, while stranded in foreign grasslands, and their task is to determine how relevant a series of items are to their situation. Following this task, a surprise memory test is given for the list of items. Memories formed in the survival scenario are superior to memories formed in control scenarios, such as imagining oneself moving to a foreign country and establishing a new life. The exact mechanisms underlying the effect are a matter of ongoing debate (for recent reviews, see Kazanas and Altarriba, 2015; Nairne and Pandeirada, 2016; Kroneisen and Erdfelder, 2017). One promising account of the effect is the richness of the encoding hypothesis (Kroneisen and Erdfelder, 2011), which proposes that the survival scenario promotes richly elaborative encoding processes, as participants think about the potential uses of an object, rendering highly distinctive and accessible memory representations. While there is increasing behavioral and neurophysiological evidence in support of the richness of encoding hypothesis (e.g., Kroneisen and Erdfelder, 2011; Fellner et al., 2013; Kroneisen et al., 2013, 2014, 2016, 2020; Röer et al., 2013; Bell et al., 2015; Kroneisen and Makerud, 2017; Forester et al., 2019, 2020), the extent to which richness of encoding during survival processing is supported or influenced by factors such as motivation is to date unclear.

Motivation can be defined as a state of desire or energy to carry out an action triggered by intrinsic (e.g., curiosity) or extrinsic (e.g., monetary reward incentive) factors (Pennartz et al., 2011; Miendlarzewska et al., 2016). Thus, an intuitive explanation of the survival processing effect is that participants are intrinsically more motivated to elaborate on items in the survival scenario. This could perhaps be due to the survival scenario being more interesting or engaging (Nairne et al., 2007). However, several studies have found survival processing effects despite control scenarios rated as equally “interesting” (Nairne and Pandeirada, 2010; Sandry et al., 2013) or “exciting” (Kang et al., 2008). Other studies, using dual-task manipulations (Kroneisen et al., 2014, 2016), have also found that while survival processing requires cognitive effort, it does not appear to be associated with increased effort relative to a moving control condition. Thus, if survival processing is more motivating, it is likely not due to perceived levels of interest in the scenario itself and likely does not exert its influence through an increase in effort. Instead, it could result in a heightened state of curiosity when evaluating the words, modulating dopaminergic reward activity in the brain (Gruber and Ranganath, 2019), or it could lead to a heightened state of physiological arousal (Löw et al., 2008).

Recent evidence for an influence of motivation comes from the finding that during encoding, survival processing is associated with greater heart rate deceleration (Fiacconi et al., 2015), an autonomic measure of the orienting response (for a review, see Bradley, 2009), which is associated with motivation to avoid harm and obtain the reward (Löw et al., 2008). Hence, survival processing may mobilize physiological and cognitive resources, which could contribute to enhanced elaboration during encoding. In light of the sparsity of direct and systematic investigations of the influence of motivation on survival processing, further examination of this possibility is thus required.

### The Effect of Motivation on Memory

To manipulate motivation, Adcock et al. (2006) presented reward cues to participants before they encoded a series of stimuli. Each cue indicated on an item-by-item basis that either a small or a large monetary reward could be earned for memorizing the upcoming stimulus. The authors found that memory was better for stimuli preceded by larger reward incentives and that this effect was associated with interactions during encoding between dopaminergic reward systems and the hippocampus. A large body of research has further substantiated the memory-enhancing effect of both extrinsic and intrinsic motivation (for reviews, see Shohamy and Adcock, 2010; Miendlarzewska et al., 2016; Gruber et al., 2019). Notably, motivation influences relatively automatic processes such as early stimulus evaluation and later memory consolidation (Miendlarzewska et al., 2016), but can also promote deeper and more active processing during encoding. For example, Cohen et al. (2014, 2016) found that motivation through reward incentive led to the adoption of more elaborative encoding strategies, as well as greater activation of prefrontal cortex areas involved in executive control and semantic elaboration.

Given the similar, adaptive effects of survival processing and reward incentive on memory, two questions arise: (1) does motivation contribute to the survival processing effect as it does to the reward incentive effect; and, (2) if so, does motivation influence the increased elaboration of survival processing? Importantly for answering the first question, there is evidence that intrinsic and extrinsic forms of motivation affect very similar neural and cognitive mechanisms (e.g., Kang et al., 2009; Düzel et al., 2010; Gruber et al., 2014; but see Duan et al., 2020), and that their effects on incidental learning are not additive (Murayama and Kuhbandner, 2011). In the study by Murayama and Kuhbandner (2011), participants were incentivized to incidentally learn the answers to trivia questions. Reward incentive during encoding improved memory for the answers to uninteresting questions but had no effect on memory for the answers to interesting questions, which participants were intrinsically motivated to learn. Hence, and in line with the finding that the addition of redundant processing effects typically has little to no added benefit to incidental learning (Paivio and Csapo, 1973; Hunt and Einstein, 1981; Burns et al., 2014), if survival processing is intrinsically more motivating, the extrinsically motivating effect of reward incentive should be reduced or absent in the survival condition.

### ERP Measures of Resource Allocation and Elaboration

Event-related potentials (ERPs) measured at the time of encoding provide a direct measure of the neurocognitive encoding mechanisms underlying behavioral effects. Two ERP components with relatively well-defined functional significances have been identified as relevant for memory encoding.

First, the P300 is a centro-parietal, positive peak that typically occurs 300–700 ms following a task-relevant stimulus (Sutton et al., 1965). The P300 increases with increasing stimulus salience, reflecting increased cognitive resource allocation during early, relatively low-level stimulus evaluation (for reviews, see Johnson, 1986; Polich, 2007), and is closely tied to memory encoding (Donchin, 1981; Fabiani et al., 1986). Further, research has shown specifically that when participants’ extrinsic motivation is high, the P300 is larger (e.g., Carrillo-de-la-Peña and Cadaveira, 2000; Goldstein et al., 2006; Elliott et al., 2020), and it can thus serve as a measure of the effect of motivation on resource allocation during low-level stimulus evaluation when other stimuli and task features are controlled for.

Second, the frontal slow wave (FSW) is a later, typically longer lasting and frontal ERP component that is thought to reflect higher-level, active control and manipulation of information within working memory (Bosch et al., 2001). The FSW occurs as a relative positivity when tasks or stimuli encourage elaborative memory encoding processes, and its amplitude is often associated with successful memory performance (Karis et al., 1984; Fabiani et al., 1990; Mecklinger and Müller, 1996; Kamp et al., 2017). The FSW can thus serve as a measure of higher-level elaboration at the time of encoding, a process generally occurring after the initial, lower-level evaluation process captured by the P300 (Fabiani et al., 1990).

In a previous study, we reported that survival processing was associated with a more positive FSW, while there was no significant effect of survival processing on the P300 (Forester et al., 2020). However, there was a tendency for increased P300, and another study did find an increase in ERP activity during survival processing with similar morphology to the P300 (Zhang et al., 2020). Therefore, while the FSW findings provide clear evidence for an increase in elaboration in the survival condition, previous ERP evidence for increased resource allocation during early stimulus evaluation (signaled by an increased P300 amplitude in the survival condition), which could indicate a general increase in motivation, is not conclusive. By manipulating motivation independently of the scenario, in the present study we were able to more carefully examine the effects of survival processing on early, low-level resource allocation (P300) and later, higher-level elaboration (FSW).

### The Present Study

In the present study, we used standard survival and moving control scenarios to manipulate survival processing, but we modified the typical encoding task in two ways. First, we adopted the choice procedure that was recently introduced by Coverdale et al. (2019), in which participants are asked to make a relative judgment between two items, rather than making an absolute judgment about a single item, in the context of the scenarios. In the present study, we asked participants to decide which of two words was more relevant to their given scenario. This not only allowed us to replicate the survival processing effect using a version of the newly introduced choice procedure, but it also provided a more natural way to include our primary modification: the reward incentive manipulation. For this manipulation, a reward cue preceded each word pair, indicating whether or not money could be earned as a result of a correct decision.

The first question we addressed was whether a difference in intrinsic motivation induced by the survival scenario relative to the moving (control) scenario contributes to the survival processing effect. We hypothesized that if this were the case, reward incentive (an extrinsic motivator) should enhance memory in the control condition (where intrinsic motivation is relatively low), but it should have a smaller or no effect in the survival condition (where intrinsic motivation is relatively high). Further, this pattern should be reflected in the P300. As a measure of resource allocation during early stimulus evaluation, the P300 is sensitive to motivation and should increase with reward incentive, but to a lesser extent in the survival condition where motivation is, according to the hypothesis, already high.

The second question was whether intrinsic motivation contributes to the active elaboration associated with survival processing and the FSW. We hypothesized that if so, reward incentive should increase the FSW, but that this effect should be reduced in the survival condition, as motivated elaboration would already be high.

To foreshadow our results, instead of the hypothesized interactions between survival processing and reward incentive, we found patterns of independent effects across all measures. We, therefore, carried out a second behavioral experiment, with a larger sample size, to test whether the effects on memory performance were truly additive rather than interactive.

## Experiment 1

### Methods

All procedures followed the ethical standards of the German Psychological Association and were approved by the ethics committee at Trier University.

#### Participants

Forty-six participants (15 males) between the ages of 18 and 33 (M = 22.27, SD = 3.65) took part in Experiment 1. They received partial course credit, plus 10 Euros as an additional financial reward (see “Procedure” section), for their participation. The data from all 46 participants were used for the behavioral and ERP analyses.

#### Stimuli

One-hundred and twenty German nouns, taken from the Berlin Affective Word List Reloaded (BAWL-R; Võ et al., 2009), was used for the encoding task. Half of the words were used in a previous survival processing study (Forester et al., 2020). All words were between four and eight letters long, were moderate in valence [*M* = 0.37, *SD* = 0.97; scale from −3 (very negative) to 3 (very positive)], arousal [*M* = 2.5, *SD* = 0.57; scale from 1 (low-arousing) to 5 (high-arousing)], and frequency (*M* = 35.9, *SD* = 104.9; appearance per million words), and were high in imageability [*M* = 6.0, *SD* = 0.33; scale from 1 (hardly imageable) to 7 (very imageable)]. An additional eight practice and four buffer words from the same list and with similar attributes were also used.

#### Scenarios and Encoding Task Instructions

We adapted the standard survival and moving scenarios that were introduced by Nairne et al. (2007), as well as the choice paradigm introduced by Coverdale et al. (2019), and added the manipulation of reward incentive on a trial-by-trial basis. The scenarios and task instructions, translated from German, were as follows.

- Survival scenario: in this task, we would like you to imagine that you are stranded in the grasslands of a foreign land, without any basic survival materials. Over the next few months, you’ll need to find steady supplies of food and water and protect yourself from predators.
- Moving (Control) scenario: in this task, we would like you to imagine that you are planning to move to a new home in a foreign land. Over the next few months, you’ll need to locate and purchase a new home and transport your belongings.
- Survival/Moving encoding task: we will show you pairs of words, and your task is to decide which of the two words would be more relevant to you in the survival/moving situation. For some word pairs, your decision will also provide an opportunity to earn bonus money. A panel of survival/moving experts has rated the relevance of each word to the situation, and for these “bonus” word pairs you will earn money if your decision matches that of the experts, allowing you to earn up to 10€ in total. You will be informed in advance which word pairs are bonus pairs, and at the end of the session, you will be paid the full amount of money that you earn. For the non-bonus word pairs, it’s up to you alone to decide which word would be more relevant in the survival/moving situation.

It should be noted that this choice procedure differed from that of Coverdale et al. (2019) in several respects. Most notably, in the present task participants decided which word was more *relevant* to the scenario *in general*, while in Coverdale et al. (2019; Exp. 1) participants decided which word was more *useful* to a *specific aspect* of the survival or moving scenario. Further, words in the present experiment were presented sequentially rather than simultaneously. These adjustments were made to facilitate comparison with our recent work (Forester et al., 2020) and to allow for the recording of ERPs elicited by single items.

#### Procedure

After participants provided informed consent and the EEG recording was prepared, participants read their respective scenarios and the encoding task instructions. They then completed the incidental-encoding task, followed by a brief distraction questionnaire, and then a surprise free-recall memory test.

For the encoding task, participants were presented with a sequence of word pairs, and their task was to decide which word from each pair was more relevant to the scenario. To manipulate reward incentive on a trial-by-trial basis, a reward cue that preceded each word pair indicated whether “bonus” money could (reward trials) or could not (non-reward trials) be earned based on their decision for that word pair (see “Scenarios and Encoding Task Instructions” section). Sixty trials (120 words) were presented in total: 30 reward trials (60 words) and 30 non-reward trials (60 words) intermixed randomly. Participants were not informed about the relative proportion of reward and non-reward trials beforehand, nor were they provided any feedback following their decision. They completed four practice trials, after which they could ask experimenter questions, as well as two buffer trials at the beginning and two at the end of the task to absorb primacy and recency effects. All stimuli were presented in size 40, Courier New font on a gray background, and two self-paced breaks were provided during the encoding task.

**Figure 1:**
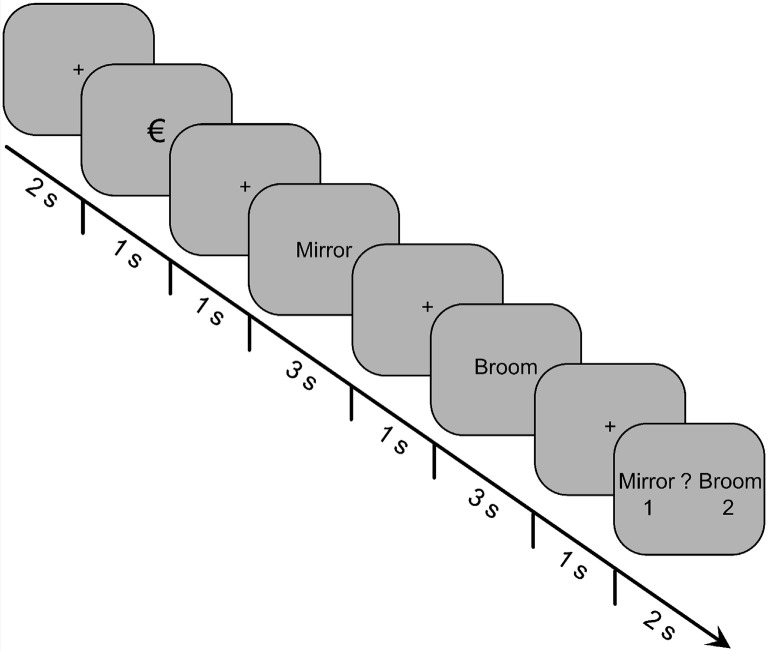
Trial structure for the incidental-encoding task of Experiment 1. Participants were presented with 60 pairs of words, and their task was to determine which word of the pair was more relevant for an imagined survival (or control) scenario. A reward cue preceded each word pair, indicating whether money could be earned based on their decision for that trial.

The depiction of an encoding-task trial is shown in Figure 1. Each trial began with a fixation cross for 2,000 ms, which was then replaced by the reward cue for 1,000 ms. The reward cue was either the “€” or “0” symbol, indicating whether it was a reward or non-reward trial, respectively. After another fixation cross was displayed for 1,000 ms, the first word of the pair was presented for 3,000 ms. The word was then replaced by a fixation cross for 1,000 ms, followed by the second word for 3,000 ms, and then another fixation cross for 1,000 ms. Finally, a decision prompt appeared for 2,000 ms, which displayed both words of the pair on either side of a question mark. The first word of a pair was always displayed to the left of the question mark, and the second word was always displayed to the right. The participants were instructed to press the “1” key if the first word was more relevant to the scenario or the “2” key if the second word was more relevant. Below each word was the number “1” or “2,” respectively, to indicate the key mapping.

After completing the encoding task, participants filled out an unrelated questionnaire for 2 min, which served as a distraction before beginning the surprise free-recall test. For the recall test, participants were given 15 min to recall as many words from the encoding task as they could. They were instructed that the order of the words, whether the words were from reward or non-reward trials, and whether the words were chosen or not during the encoding task, was irrelevant for the recall task. Participants submitted words by typing them onto the screen using a keyboard and then pressing the “enter” key. After a word was submitted it could no longer be edited or deleted, but all submitted words were continuously visible on the screen throughout the test.

Following the recall test, the EEG cap was removed, and the participants were debriefed. Because there were not expert judges that rated the relevance of the word stimuli to the survival/moving scenarios, and thus there were no correct or incorrect decisions, all participants were paid the full 10€ in bonus money.

#### Design

A 2 × 2 (Scenario × Reward) mixed factor design was used, with Scenario (survival vs. control) as the between-subjects factor and Reward (reward vs. non-reward) as the within-subjects factor. Half of the 46 participants were randomly assigned to the survival scenario and half to the control scenario.

We conducted a sensitivity analysis using G*Power 3.1.9.4 (Faul et al., 2009) to determine the effect sizes for which 2 × 2 mixed factor ANOVAs on the behavioral measures were sufficiently sensitive. With 46 participants, assuming *α* = 0.05, 1 − β = 0.8 and a correlation of between repeated measurements, the *F*-tests were sensitive to small to medium effects (*f* = 0.21) of Reward and the Reward × Scenario interactions, and medium to large main effects of Scenario (*f* = 0.36).

#### Behavioral Memory Analysis

Memory recall performance was calculated as the proportion of correctly recalled words per condition. Words were counted as correctly recalled if they exactly matched one of the words presented during the encoding task, or if they were considered an insignificant misspelling or variation (e.g., the pluralization of a singular word) by research assistants who were unaware of which Scenario and Reward conditions the words pertained to. Any complete words that were not counted as correctly recalled and were not part of the practice or buffer trials were counted as false memory intrusions.

#### EEG Recording and Analysis

Continuous EEG was recorded using 33 Ag/AgCL electrodes (Fp1/2, F7/8, F3/4, Fz, FC5/6, FC1/2, FCz, T7/8, C3/4, Cz, CP5/6, CP1/2, TP9/10, P7/8, P3/4, Pz, PO9/10, O1/2, and Iz) according to the extended 10/20 system. Electrode FCz was the online reference electrode and the ground electrode was at Az. A NeuroOne amplifier (Bittium Corporation, Finland) amplified the EEG signal online from 0.16 to 7,000 Hz with an analog 125 Hz low-pass filter and digitized at a rate of 500 Hz. BrainVision Analyzer 2.1 (Brain Products, Inc.) was used to analyze the EEG data offline. First, the data were re-referenced to linked mastoids (TP9/10), and the signal at FCz was mathematically reconstructed. The data were then filtered using a 30 Hz low-pass IIR Butterworth filter with a 24 dB/octave roll-off and a 50 Hz notch IIR Butterworth filter with a 72 dB/octave roll-off. The EEG was segmented from 500 ms before to 3,000 ms after word onset for the initial, individual presentation of both the first and second word of a pair. Ocular artifacts were corrected semi-automatically using the infomax (Bell and Sejnowski, 1995) independent component analysis algorithm in BrainVision Analyzer. Segments with remaining artifacts were then automatically removed whenever the segment contained a 30 μV or larger amplitude change in a time window of 1 ms, or an absolute amplitude difference of more than 120 μV within the segment. For each participant, artifact-free EEG segments were averaged, separately for reward and non-reward words, and baseline corrected using the 500 ms before word onset. A mean of 57 trials (minimum = 44) was included in the participant-ERP averages for reward words and a mean of 57 trials (minimum = 38) for non-reward words.

We measured ERP amplitude at nine electrodes (F3, Fz, F4, C3, Cz, C4, P3, Pz, and P4), forming a 3 (Anteriority) by 3 (Laterality) spatial grid, as this allowed us to encompass the typical centro-parietal distribution of the P300 (Spencer et al., 2001) and the typical fronto-central distribution of the slow-wave (Bosch et al., 2001; Kamp et al., 2019). For these electrodes, we examined mean ERP activity within three time windows. The first time window, from 500 to 700 ms, was used to capture the P300 (e.g., Fabiani et al., 1990; Kamp et al., 2016; Forester et al., 2020). The second time window, from 800 to 1,200 ms, was used to capture the early, relatively steep, slow-wave activity that was maximal at approximately 1,000 ms in our previous study of survival processing during encoding (Forester et al., 2020; see also, e.g., Fabiani et al., 1990; Mecklinger and Müller, 1996; Höltje et al., 2019). The third time window, from 1,200 to 2,000 ms, was used to capture the typical sustained slow-wave activity (Kamp et al., 2017; 2019) which was also observed in our previous study (Forester et al., 2020). The suitability of these time windows, henceforth referred to as the “P300,” “early slow-wave,” and “late slow-wave” time windows, respectively, was confirmed by visual inspection of the present, grand average ERP waveforms.

#### Statistical Analysis

Behavioral recall performance was analyzed statistically using a 2 × 2 (Scenario × Reward) mixed factor ANOVA. The number of false memory intrusions between the survival and moving conditions was compared using an independent samples *t*-test. For the ERP results, the mean ERP amplitude within each time window was compared statistically using 2 × 2 × 3 × 3 (Scenario × Reward × Anteriority × Laterality) mixed factor ANOVAs. The two electrode factors (Anteriority and Laterality) were included to explore potential differences in scalp distributions. However, to avoid alpha-error accumulation, significant effects that included the electrode factors were only reported if they qualified significant effects of Scenario, Reward, or a Scenario × Reward interaction. We used a Greenhouse-Geisser correction whenever the ANOVA assumption of sphericity was violated.

### Results

#### Behavioral Recall Results

**Figure 2:**
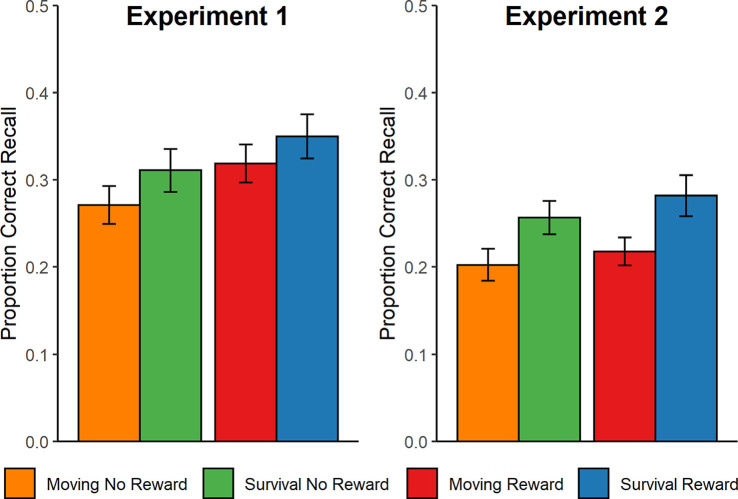
Behavioral recall performance in Experiments 1 (left) and 2 (right). Performance is calculated as the proportion of correctly recalled words per condition (30 words per condition). Error bars represent the standard error of the mean. ERPs were recorded during Experiment 1.

Descriptively (see Figure 2), more words were recalled from reward trials than non-reward trials, more words were recalled from the survival compared to the control scenario, and there did not appear to be an interaction between the two factors. The statistical analysis confirmed a significant main effect of Reward on the proportion of correctly recalled words, *F*_(1,44)_ = 10.25, *p* = 0.003, $\eta_{\text{p}}^{2}$ = 0.19, but the main effect of Scenario on recall was not significant, *F*_(1,44)_ = 1.38, *p* = 0.247, $\eta_{\text{p}}^{2}$ = 0.03. No evidence was found for an interaction, *F*_(1,44)_ = 0.1, *p* = 0.75, $\eta_{\text{p}}^{2}$ = 0.00. There were significantly more false memory intrusions associated with the moving scenario (M = 3.1, SD = 3.7) compared to the survival scenario (M = 1.2, SD = 1.1), *t*_(44)_ = 2.43, *p* = 0.019, *d* = 0.72.

#### ERP Results

**Figure 3:**
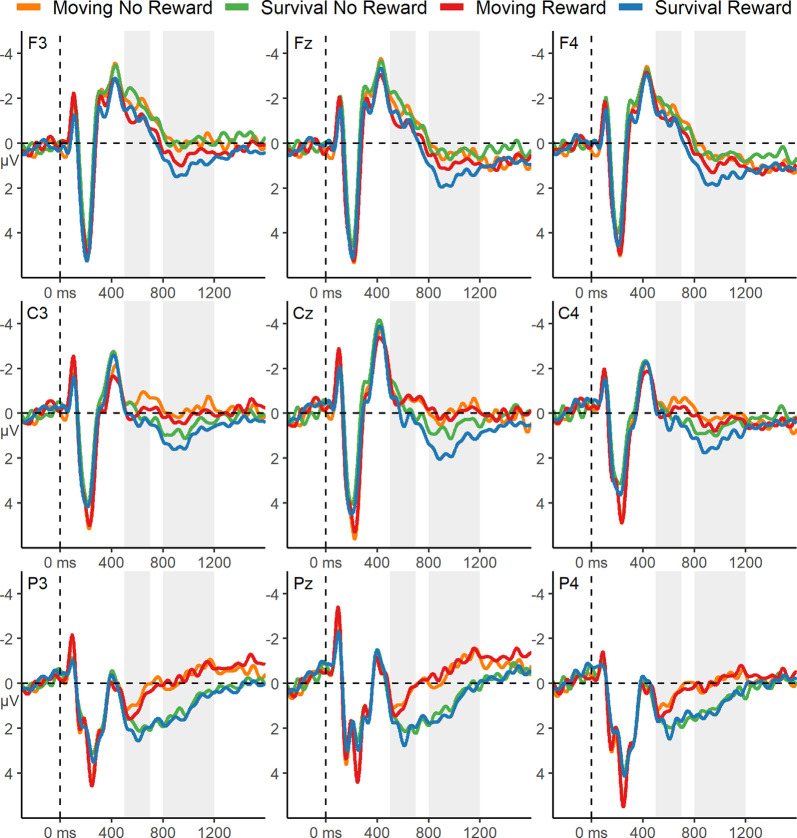
ERPs from 500 ms before, to 1,600 ms after, word onset during encoding. The gray boxes highlight the P300 (500–700 ms) and the early slow-wave (800–1,200 ms) time windows. The ERPs are displayed with a 12 Hz low-pass filter.

Grand average ERPs elicited by words during encoding is displayed in Figure 3. Visual inspection of these waveforms revealed clear P300 and early slow-wave components. Both reward incentive and the survival scenario appeared to be associated with a more positive P300, but there was no indication of an interaction. Both reward incentive and survival processing also appeared to be associated with increased early FSW activity, with no evidence for a reduced effect of reward incentive in the survival condition.

##### P300 Time Window

Statistical analysis of the encoding ERPs during the P300 time window revealed a significant main effect of reward, *F*_(1,44)_ = 5.18, *p* = 0.028, $\eta_{\text{p}}^{2}$ = 0.11, indicating a more positive P300 elicited by words on reward trials. By contrast to the visual impression (Figure 3), there was no significant main effect of Scenario, *F*_(1,44)_ = 0.54, *p* = 0.47, $\eta_{\text{p}}^{2}$ = 0.01. There was also no significant Scenario × Reward interaction *F*_(1,44)_ = 0.002, *p* = 0.97, $\eta_{\text{p}}^{2}$ < 0.01.

##### FSW Time Windows

There was a significant main effect of Reward on the early slow wave, *F*_(1,44)_ = 8.21, *p* = 0.006, $\eta_{\text{p}}^{2}$ = 0.16, indicating a more positive early slow wave elicited during encoding for reward trials. This effect was qualified by a significant Reward × Anteriority interaction *F*_(1.23,54.06)_ = 8.8, *p* = 0.003, $\eta_{\text{p}}^{2}$ = 0.17, reflecting that the Reward effect was largest at fronto-central electrodes (Figure 3). There was also a significant main effect of Scenario, *F*_(1,44)_ = 5.38, *p* = 0.025, $\eta_{\text{p}}^{2}$ = 0.11, indicating a more positive early slow wave in the survival condition. There was no significant Reward × Scenario interaction during the early slow wave time window, *F*_(1,44)_ = 2.01, *p* = 0.16, $\eta_{\text{p}}^{2}$ = 0.05. Moreover, there were no significant effects during the late slow wave time window (all *p*-values >0.23).

## Experiment 2

Based on a hypothesized role of motivation, we predicted that the effects of survival processing and reward incentive on behavioral memory performance would interact, reflecting a diminished effect of extrinsic motivation in the (presumably more intrinsically motivating) survival condition. In contrast to our prediction, we found a pattern descriptively supporting additive effects, with no interaction. However, because the main effect of survival processing on memory performance was not statistically significant (even when tested within each reward condition), it is unclear if an effect was not detected due to insufficient power (for standard error probabilities α = β = 0.05, the *F*-tests were only sensitive to fairly large between-subject effects: *f* ≥ 0.36), or if instead, some peculiarity of the encoding task eliminated the survival processing effect. For example, the sequential presentation of the word pairs in a comparative relevance rating task may have increased working memory load compared to the typical paradigm, thus reducing the ability for rich encoding and hence the survival processing effect (Kroneisen et al., 2014, 2016). We thus conducted a follow-up behavioral study with a larger sample size that was sufficiently powered to detect both main and interaction effects of survival processing and reward incentive on recall performance. Note that since the results of Experiment 2 are vital for a proper interpretation of the ERP results of Experiment 1, we will postpone a discussion of the ERP patterns to the general discussion.

### Methods

Experiment 2 was conducted online, using PsyToolkit (Stoet, 2010, 2017), with student participants from the University of Trier. EEG was not recorded.

#### Participants

Ninety-eight participants (22 males) between the ages of 18 and 35 (M = 22.6, SD = 2.75) received partial course credit for their participation in Experiment 2.

#### Scenarios and Instructions

Because the experiment was conducted online, we modified reward manipulation. Instead of reward trials providing the opportunity to earn cash, participants instead had the opportunity to earn money that would be donated by the research team to a local charity. Note that although a cash reward and a charitable donation are not identical, they have both been shown to be effective extrinsic motivators and to have the same effect on the brain’s reward system (Moll et al., 2006). At the beginning of the experiment, the charity and the opportunity to earn money for that charity were briefly described. The same survival and moving (control) scenarios from Experiment 1 were then presented, but the reward manipulation instructions were altered slightly. The relevant section read as follows: “…*for these “bonus” word pairs you will earn money if your decision matches that of the experts, allowing you to earn up to 10€ in total. At the end of the study, 20% of the money that you, along with all other participants in the study, earn, will be paid to the [name of charity]. For the non-bonus word pairs, it’s up to you alone to decide which word would be more relevant in the survival/ moving situation.”*

#### Procedure and Tasks

Participants began the experiment with the incidental-encoding task, in which they decided which word from randomly formed pairs was more relevant to the scenario. The task, stimuli, and reward cues were the same as in Experiment 1, with one primary exception: The two words of a pair were presented side by side for 5 s, rather than sequentially, therefore aligning with previous behavioral studies (Coverdale et al., 2019). During these 5 s, participants made their decision by clicking on a word with the mouse cursor. The two words remained on the screen until the full 5 s had passed, and then the next trial began.

After completing the encoding task, and a 2-min distraction questionnaire, participants began the surprise free recall task. This differed from the recall task of Experiment 1 only in that the words did not remain on the screen after being submitted.

#### Design

The same 2 × 2 (Scenario × Reward) mixed factor design was used as in Experiment 1, with half of the subjects being randomly assigned to the survival scenario and the other half to the control scenario. We recruited 98 participants based on an *a priori* power analysis for detecting a medium-sized (*f* = 0.25) main effect of Scenario (Scofield et al., 2017), assuming *α* = 0.05, 1 − β = 0.8, and α = 0.50 between repeated measurements. This sample size provided sensitivity for detecting small effects (*f* = 0.14) of Reward and Scenario × Reward. The power analyses were conducted using G*Power 3.1.9.4 (Faul et al., 2009).

### Results

Using the same procedure as in Experiment 1, recall performance was calculated as the proportion of correctly recalled words within each condition and analyzed statistically using a 2 × 2 (Scenario × Reward) mixed factor ANOVA. The ANOVA revealed significant main effects of Scenario, *F*_(1,96)_ = 4.69, *p* = 0.032, $\eta_{\text{p}}^{2}$ = 0.05, and Reward, *F*_(1,96)_ = 5.18, *p* = 0.025, $\eta_{\text{p}}^{2}$ = 0.05, reflecting that both the survival scenario and reward incentive were associated with improved memory recall (Figure 2). Again, however, there was no interaction, *F*_(1, 96)_ = 0.52, *p* = 0.47, $\eta_{\text{p}}^{2}$ < 0.01. Furthermore, an independent samples *t*-test revealed no significant difference in the number of false memory intrusions between the moving (M = 2.0, SD = 2.1) and survival (M = 1.9, SD = 1.7) scenarios, *t*_(96)_ = 0.32, *p* = 0.747, *d* = 0.07.

## Bayesian Analysis: Evidence Against an Interaction on Memory Performance

In both Experiments 1 and 2, we found no evidence for an interaction between survival processing and reward incentive on memory performance. However, because these null findings do not necessarily reflect the absence of an interaction, we conducted an additional Bayesian analysis using JASP (JASP Team, 2019; version 0.11.1) to assess whether there was evidence for or against interaction between survival processing and reward incentive on behavioral memory performance. To do this, we first computed *z*-scores of the memory performance data for each experiment, and then conducted a Bayesian ANOVA on the *z*-scores, collapsed across experiments. Using the Bayes Factor, we compared a (null) model that contained the main effects of Reward and Scenario against a model that also included their interaction and found evidence against the interaction, thus supporting the additive effects null model (BF_01_ = 5.88)^1^.

## Supplemental Analysis: The Effect of Choice on Memory Performance

The choice procedure of the encoding task additionally allowed us to explore how the effect of choice (i.e., comparing chosen vs. unchosen words; Coverdale and Nairne, 2019; Coverdale et al., 2019) interacts with reward incentive and survival processing to affect memory performance. We thus conducted supplemental 2 × 2 × 2 (Scenario × Reward × Choice) mixed factor ANOVAs on recall performance. In both experiments, we found significant main effects of Choice (Exp. 1: *F*_(1,44)_ = 119.18, *p* < 0.001, $\eta_{\text{p}}^{2}$ = 0.73; Exp. 2: *F*_(1,96)_ = 127.13, *p* < 0.001, $\eta_{\text{p}}^{2}$ = 0.57), such that recall was better for chosen words (Exp. 1: M = 22.6; Exp. 2: M = 18.5) compared to unchosen words (Exp. 1: M = 14.6; Exp. 2: M = 11.3). The effect of choice did not significantly interact with Reward (Exp. 1: *F*_(1,44)_ = 0.007, *p* = *0*.934, $\eta_{\text{p}}^{2}$ < 0.01; Exp. 2: *F*_(1, 96)_ = 2.01, *p* = 0.152, $\eta_{\text{p}}^{2}$ = 0.02) or Scenario (Exp. 1: *F*_(1,44)_ = 1.95, *p* = 0.17, $\eta_{\text{p}}^{2}$ = 0.04; Exp. 2: *F*_(1,96)_ = 1.54, *p* = 0.22, $\eta_{\text{p}}^{2}$ = 0.02) in either experiment, nor was there a significant three-way interaction (Exp. 1: *F*_(1,44)_ = 0.61, *p* = *0*.44, $\eta_{\text{p}}^{2}$ = 0.01; Exp. 2: *F*_(1,96)_ = 0.14, *p* = *0*.71, $\eta_{\text{p}}^{2}$ < 0.01).

## Discussion

We manipulated extrinsic motivation *via* reward incentive orthogonally to survival processing and hypothesized that if intrinsic motivation contributed substantially to the survival processing effect, then the effect of extrinsic motivation should be reduced in the survival condition. In opposition to this hypothesis, we found evidence for additive effects of survival processing and reward incentive, suggesting that motivation is unlikely to play a major role in the survival processing effect. Further, although the ERP evidence suggests that survival processing and extrinsic motivation can both influence elaboration during encoding (see “FSW: Independent Effects of Survival Processing and Reward Incentive on Elaboration” section), we did not find evidence that motivation explains the elaboration associated with the survival processing effect.

### Memory Performance: Intrinsic Motivation Does Not Underlie the Survival Processing Effect

In both Experiments 1 and 2, we found no evidence that the enhancing effect of reward incentive on memory performance was diminished in the survival condition, and we instead found a pattern of additive effects. The absence of interaction was supported by a Bayesian analysis across the two experiments. It should be noted that the type of reward incentive (cash vs. charitable donation) differed between the two experiments, possibly leading to differences in the way or the degree to which, the reward was processed between them. However, since both rewards led to enhanced memory in the present study, and previous work has shown that the two types of reward affect neural reward systems in the same way (Moll et al., 2006), it appears relatively safe to conclude that survival processing and reward incentive exert independent effects on memory.

Adding extrinsic motivation to an intrinsically motivating task can lead to additive (Cerasoli et al., 2014) or undermining (Deci et al., 1999; Murayama et al., 2010) effects of extrinsic and intrinsic motivation on general behavioral performance, and additive effects on intentional memory formation (Duan et al., 2020). However, the effect of extrinsic motivation on incidental learning is reduced or eliminated when intrinsic motivation is already high (Murayama and Kuhbandner, 2011) and redundant processing effects on incidental learning are usually not additive (Paivio and Csapo, 1973; Hunt and Einstein, 1981). Therefore, the presently observed additive effects provide evidence against the hypothesis that the survival processing effect depends on greater intrinsic motivation.

Because there has been little direct investigation into the role of motivation in the survival processing effect, these findings offer an important step towards ruling out motivation as a crucial factor and should be replicated using different control conditions and tasks. However, the results are somewhat inconsistent with the finding that an autonomic measure of the orienting response was increased in the survival condition, with this increase being associated with the memory performance advantage (Fiacconi et al., 2015). Together, these findings could suggest that while neither higher-level motivation (the present study) nor subjective emotional arousal (Nairne et al., 2007; Otgaar et al., 2010; Soderstrom and McCabe, 2011; Smeets et al., 2011; Bell et al., 2013, 2015; Yang et al., 2014) is likely to be crucial in the survival processing effect, a contribution of lower-level, more automatic processes related to physiological resource allocation may play a role. This idea is generally in keeping with the presumed evolutionary origin of the effect, and the idea will be returned to in the following section on the P300 effects.

Finally, while there was almost no difference in the number of false memory intrusions between the survival and moving scenarios in Experiment 2, the moving scene was associated with more intrusions in Experiment 1, an effect that we have observed previously (Forester et al., 2020). The reason for this discrepancy is unclear, but in either case, these findings suggest that the survival processing effect is unlikely to result from a factor that simply increases the number of recall attempts, or that increases both correct and false memories simultaneously (e.g., Otgaar and Smeets, 2010; see Forester et al., 2020 for further discussion).

### P300: Reward Incentive Increases Resource Allocation Independently of Survival Processing

Reward incentive increased the P300, indicating that it motivated an increase in resource allocation for the early evaluation of words during encoding. If survival processing leads to a relatively high degree of intrinsic motivation, we should have observed a reduction in this effect of extrinsic motivation on the P300 in the survival condition. Instead, we did not find any difference in the degree to which reward incentive increased the P300 in the survival and control conditions. The lack of interaction is unlikely to be due to a lack of power, as we had sufficient power for detecting even small interaction effects, and descriptively there was no suggestion of interaction (Figure 3). This pattern is in line with the behavioral memory performance findings, further suggesting that a difference in motivation is unlikely to contribute to the survival processing effect.

Interestingly, we observed a conspicuous trend for an increased overall P300 in the survival condition compared to the moving condition, independent of the reward manipulation (Figure 3), consistent with a similar trend in our previous study (Forester et al., 2020) and with another recently published study (Zhang et al., 2020). Therefore, though the difference was not statistically reliable in the present study, a potential effect of survival processing on the P300 remains to be investigated in future studies, because it could be that, mirroring the nonsignificant survival processing effect on behavioral memory performance in Experiment 1, the present study lacked the power to detect such a main effect. If future research substantiated a difference between the survival and the control condition in overall P300 amplitude, this would suggest that resource allocation to the early evaluation of words is affected by the survival scenario. However, the present results suggest that this difference would not be due to differences in intrinsic motivation, given that the effect of extrinsic motivation was not affected by the survival scenario. Consistent with findings by Fiacconi et al. (2015), this pattern would rather suggest that differences in physiological arousal might affect early, lower-level resource allocation during survival processing, which could contribute to the memory enhancement effect independently of motivation. If borne out, this would have potentially important implications, as it could suggest that the survival-processing task leads to physiological effects akin to those produced in response to an actual survival situation (Bradley, 2009).

In sum, the P300 effects failed to support an influence of intrinsic motivation on the survival processing effect, thus aligning with the behavioral memory performance findings.

### FSW: Independent Effects of Survival Processing and Reward Incentive on Elaboration

Both survival processing and reward incentive were associated with significantly greater positivity during the early FSW time window. The former effect is consistent with our previous ERP findings showing that survival processing increases the FSW measure of elaboration (Forester et al., 2020) and supports the richness of the encoding hypothesis (Kroneisen and Erdfelder, 2011). The latter effect indicates that extrinsic motivation can also lead to increased elaboration during incidental encoding (Cohen et al., 2014). The factors did not interact, indicating that survival processing and motivation independently increase elaboration. Taken together with the finding that the survival processing effect on memory performance did not depend on motivation, the hypothesis that motivation is responsible for the enhanced elaboration of survival processing is not supported by the present results.

Notably, while the early slow-wave effect of reward incentive was restricted to frontal scalp locations, the slow-wave activity associated with survival processing was widespread and extended to parietal electrodes. It has been shown that the scalp distribution of slow-wave activity can vary with the content of information held in working memory (e.g., verbal, visual, or spatial, Bosch et al., 2001; Khader et al., 2007). Accordingly, one possible explanation for the extended scalp distribution of the effect is that survival processing leads to elaboration across more widespread neural, and perhaps functional, domains than elaboration that is stimulated by reward incentives. This would be consistent with previous research showing that survival processing increases communication between distant cortical areas during encoding (Fellner et al., 2013) and recruits a more widespread network of cortical areas during memory retrieval (Forester et al., 2019).

Another possibility is that the parietal aspect of the slow-wave activity observed in the present study reflects a separate process that is also affected by survival processing. For example, a component called the late positive potential (LPP), which is typically elicited by emotional stimuli (for a review, see Hajcak et al., 2010), has a very similar morphology to the present parietal activity. The LPP is thought to reflect sustained attentional engagement to emotional information, and this activity could suggest that the survival scenario led to increased emotional reactivity (Hajcak et al., 2014). However, the LPP is often modulated by reward (for a review, see Glazer et al., 2018), while there was no effect of reward incentive on the slow wave at parietal electrodes in the present study. Although the ultimate functional significance of the posterior aspect of the slow-wave activity observed in the survival condition of the present study remains a matter of speculation, the finding nonetheless emphasizes a dissociation between the neural mechanisms engaged by elaboration stimulated by survival processing and extrinsic motivation.

It is also worth highlighting that, to our knowledge, this is the first study to demonstrate that motivation *via* reward incentive can increase the FSW measure of elaborative encoding. This is at odds with a recent study that did not find evidence that reward incentive increases the FSW (Elliott et al., 2020). However, several differences between these two studies could explain the discrepancy. Most notably, an elaborative encoding task was not used in the study by Elliott et al. (2020), while the relevance rating task of the present study is likely highly elaborative, and the FSW associated with item encoding is generally only elicited by elaborative or associative tasks (Fabiani et al., 1990). Further, the present finding is consistent with work by Cohen et al. (2014, 2016), who did find that elaborative encoding strategies and related fMRI activity in the prefrontal cortex varied with extrinsic motivation. Future studies should therefore further pursue the possibility that qualitative shifts in the manner of encoding contribute to the enhancing effect of motivation on memory during incidental encoding.

A final point of discussion is that we did not observe any effects during the late slow wave time window, while the FSW commonly extends into this and even later time windows (e.g., Bosch et al., 2001; Kamp et al., 2019). Though we did observe late FSW activity associated with survival processing in our previous study (Forester et al., 2020), the earlier effect was larger and closely matched the present early FSW effect, suggesting that this early activity may be more consistently elicited and hence perhaps more relevant to the survival processing effect. It is important to note though that we lacked sufficient power for detecting small to medium-sized main effects of survival processing in Experiment 1, and thus the absence of such effects on ERP activity could alternatively reflect this lack of power. In either case, future research is necessary to determine whether the relatively early and late slow-wave activity reflect functionally distinct processes, or only a shift in timing or duration, and the extent to which the timing of FSW effects contributes to the survival processing effect.

### The Choice Procedure and the Choice Effect

To our knowledge, the present study is the first to independently replicate the survival processing effect using a choice procedure (Coverdale et al., 2019), further establishing the generalizability of the effect. Consistent with Coverdale et al. (2019), we also found that chosen words were better remembered and that this effect did not significantly interact with survival processing. These results, therefore, help to confirm that the survival processing effect does not depend on the congruence or fit of an item to the scenario when comparative judgments are made, nor on participants having chosen an item (Coverdale and Nairne, 2019). We further found that the choice effect did not significantly interact with reward incentive, perhaps suggesting that the mnemonic benefit of choice does not stem from the perceived value of an item during encoding (Chakravarty et al., 2019; Coverdale and Nairne, 2019). However, it should be noted that the relationship between choice and reward was not of primary focus in the present study, and future research will be required to confirm this tentative suggestion of their independence.

## Conclusions

Despite the acknowledgment of motivation as a potential (confounding) factor in the survival processing effect since its discovery, direct evidence either for or against its influence has largely been missing. Across two experiments in which we independently manipulated survival processing and motivation through reward incentive, we found no evidence to support the role of motivation in the survival processing effect. Further, an examination of electrophysiological activity revealed that the effects of survival processing and extrinsic motivation during encoding are independent. These findings, therefore, indicate that while the adaptive, memory-enhancing effects of survival processing and motivation for reward may rely on overlapping neural mechanisms, the survival processing effect is unlikely to be due to motivation.

## Data Availability Statement

The data are available at https://osf.io/tdyrb/.

## Ethics Statement

The studies involving human participants were reviewed and approved by University of Trier Ethics Committee. The patients/participants provided their written informed consent to participate in this study.

## Author Contributions

GF and S-MK participated in the study design, discussion of the data, and writing of the manuscript. GF lead data collection and analysis. All authors contributed to the conceptual development of the study and critically revised the manuscript. All authors contributed to the article and approved the submitted version.

## Conflict of Interest

The authors declare that the research was conducted in the absence of any commercial or financial relationships that could be construed as a potential conflict of interest.

## Footnotes

^1^The same result was also found when only considering the data from Experiment 2 (BF_01_ = 3.47).
